# Real-world assessment of Fluorecare SARS-CoV-2 Spike Protein Test Kit

**DOI:** 10.1515/almed-2021-0041

**Published:** 2021-05-24

**Authors:** Gian Luca Salvagno, Gianluca Gianfilippi, Laura Pighi, Simone De Nitto, Brandon M. Henry, Giuseppe Lippi

**Affiliations:** Section of Clinical Biochemistry, University of Verona, Verona, Italy; Service of Laboratory Medicine, Pederzoli Hospital, Peschiera del Garda, Italy; Medical Direction, Pederzoli Hospital, Peschiera del Garda, Italy; The Heart Institute, Cincinnati Children’s Hospital Medical Center, Cincinnati, OH, USA

**Keywords:** COVID-19, diagnosis, immunoassay, laboratory medicine, SARS-CoV-2

## Abstract

**Objectives:**

Since commercial SARS-CoV-2 (severe acute respiratory syndrome coronavirus 2) antigen rapid detection tests (Ag-RDTs) display broad diagnostic efficiency, this study aimed to evaluate the clinical performance of Fluorecare SARS-CoV-2 Spike Protein Test Kit in a real-life scenario.

**Methods:**

The study population consisted of a series of patients undergoing SARS-Cov-2 diagnostic testing at Pederzoli Hospital of Peschiera del Garda (Verona, Italy). A nasopharyngeal swab was collected upon hospital admission and assayed with molecular (Altona Diagnostics RealStar^®^ SARSCoV-2 RT-PCR Kit) and antigen (Fluorecare SARS-CoV-2 Spike Protein Test Kit) tests.

**Results:**

The study population consisted of 354 patients (mean age, 47 ± 20 years; 195 women, 55.1%), 223 (65.8%) positive at molecular testing. A significant correlation was found between Fluorecare SARS-CoV-2 Spike Protein Test Kit and Altona (both *S* and *E* genes: r=−0.75; p<0.001). The cumulative area under the curve in all nasopharyngeal samples was 0.68. At ≥1.0 S/CO manufacturer’s cut-off, the sensitivity, specificity, negative and positive predictive values were 27.5, 99.2, 41.5 and 98.5%, respectively. Considerable improvement of sensitivity was observed as Ct values decreased, becoming 66.7% in samples with mean Ct values <30, 90.5% in those with mean Ct values <25, up to 100% in those with mean Ct values <20.

**Conclusions:**

The modest sensitivity and negative predictive value of Fluorecare SARS-CoV-2 Spike Protein Test Kit makes unadvisable to use this assay as surrogate of molecular testing for definitively diagnosing SARS-CoV-2 infection, though its suitable sensitivity at high viral load could make it a reliable screening test for patients with higher infective potential.

## Introduction

Due to the huge pressure imposed on routine diagnostic laboratories by the ongoing coronavirus disease 2019 (COVID-19) pandemic outbreak, rapid antigen tests hold premise as suitable alternatives for widespread, rapid and efficient identification of subjects with severe acute respiratory syndrome coronavirus 2 (SARS-CoV-2) infection [1]. Nonetheless, as recently endorsed by both the World Health Organization (WHO) [2], and by the Task Force on COVID-19 of the International Federation of Clinical Chemistry and Laboratory Medicine (IFCC) [3], the clinical performance of each SARS-CoV-2 antigen rapid detection test (Ag-RDT) must be thoroughly evaluated before its introduction – at whatever stage – into clinical practice. Therefore, this work was aimed to assess the clinical performance of Fluorecare SARS-CoV-2 Spike Protein Test Kit in a real-life scenario.

## Materials and methods

### Study population

Our study population consisted of a series of patients undergoing SARS-Cov-2 diagnosis at the Laboratory Medicine Service of the Pederzoli Hospital (Peschiera del Garda, Verona, Italy) between April 2 and 19, 2021, for being either symptomatic or in close contact with COVID-19 cases. A nasopharyngeal swab (Virus swab UTM™, Copan, Brescia, Italy) was immediately collected upon hospital admission and assayed with both molecular and antigen testing.

### Molecular testing

SARS-CoV-2 nucleic acid amplification testing (NAAT) was carried out using Altona Diagnostics RealStar^®^ SARSCoV-2 RT-PCR Kit (Altona Diagnostics GmbH, Hamburg, Germany). This real-time reverse transcription polymerase chain reaction (rRT-PCR) entails two separate amplifications and detections, independently targeting the SARS-CoV-2 *E* and *S* gene sequences. A probe and a primer set for internal control is also included in the test kit, for detecting possible rRT-PCR inhibition. The test was carried out on a Bio-Rad CFX96™ Deep Well Dx Real-Time PCR Detection System (Bio-Rad Laboratories, Hercules, CA, USA). Results were considered positive when the cycle threshold (Ct) values of both *S* and *E* SARS-CoV-2 genes were lower than 45.

### Antigen testing

Antigen testing was carried out using the immunochromatographic assay Fluorecare SARS-CoV-2 Spike Protein Test Kit (Microprofit Biotech, Shenzhen, China). Briefly, patient nasopharyngeal sample is mixed with a solution containing fluorescently-labelled anti-SARS-CoV-2 spike protein antibodies and deposited in the sample window of the test kit. The eventually generated immunocomplexes migrate along a nitrocellulose membrane up to the detection area, where they generate a red line (in SARS-CoV-2 spike protein-positive samples). The fluorescently-labelled anti-SARS-CoV-2 spike protein antibodies also migrate towards a quality control window, where another line is generated to attest that the kit is working properly. The full test can be completed within 15–30 min. A dedicated point of care instrumentation can be used for quantitative fluorescence reading (Fluorecare MF-T1000; Microprofit Biotech, Shenzhen, China), which is then reported as arbitrary measuring unit (i.e., signal to cut-off; S/CO). The test is considered positive when the S/CO value is ≥1.0. According to the manufacturer, the percent positive and negative agreement vs. a reference RT-PCR technique is 92.2 and 100%, respectively.

### Statistical analysis

In our study, the diagnostic performance of Fluorecare SARS-CoV-2 Spike Protein Test Kit vs. the reference NAAT was evaluated with Spearman’s correlation, by constructing receiver operating characteristic (ROC) curves, and calculating the diagnostic sensitivity, specificity, negative predictive value (NPV) and positive predictive value (PPV). Statistical analysis was performed with Analyse-it software (Analyse-it Software s., Leeds, UK). This investigation was carried out as part of clinical laboratory operations, using pre-existing specimens collected for routine SARS-CoV-2 diagnostics at the local facility, and thereby patient informed consent and Ethical Committee approval were unnecessary. All test results were anonymized prior to statistical analysis. The study was conducted in accordance with the Declaration of Helsinki, under the terms of relevant local legislation.

## Results

The final study population consisted of 354 patients (mean age, 47 ± 20 years; 195 women, 55.1%), 223 of whom (65.8%) were positive at NAAT (i.e., Ct values of both SARS-CoV-2 *S* and *E* genes <45). The Ct values of positive samples were 29.8 ± 7.1 and 30.3 ± 7.0 for the SARS-CoV-2 *S* and *E* genes, respectively. A highly significant Spearman’s correlation was found between values of Fluorecare SARS-CoV-2 Spike Protein Test Kit and measurable Ct values of *S* (r=−0.75; 95% CI, −0.80 to −0.69; p<0.001) and *E* (r=−0.75; 95% CI, −0.80 to −0.68; p<0.001) genes.

The overall diagnostic performance of Fluorecare SARS-CoV-2 Spike Protein Test Kit, as well as performance stratified according to Altona Ct values, is summarized in Table 1. The cumulative area under the curve (AUC) in all nasopharyngeal samples was 0.68. At the ≥1.0 S/CO manufacturer’s cut-off, the sensitivity, specificity, NPV and PPV values were 27.5, 99.2, 41.5 and 98.5%, respectively. The best cut-off calculated from the ROC curve was 0.18 S/CO, associated with 42.9% (95% CI, 36.5–49.5%) sensitivity, 92.6% (95% CI, 86.4–96.5%) specificity, 45.7% (95% CI, 42.7–48.8%) NPV and 91.7% (95% CI, 85.4–95.5%) PPV. Nonetheless, considerable improvement of diagnostic sensitivity was observed as the Ct values decreased, increasing to 66.7% in samples with mean Ct values <30, 90.5% in those with mean Ct values <25, up to 100% in those with very high viral load (i.e., mean Ct values <20).

**Table 1:** Clinical performance of Fluorecare SARS-CoV-2 Spike Protein Test Kit stratified according to cycle threshold (Ct) values.

Ct values	n	AUC	SN	SP	NPV	PPV
All samples	354	0.68 (95% CI, 0.62–0.73)	27.5% (95% CI, 21.8–33.7%)	99.2% (95% CI, 95.5–100%)	41.5% (95% CI, 39.6–43.5%)	98.5% (95% CI, 90.0–99.8%)
<30	96	–	66.7% (95% CI, 56.3–76.0%)	–	–	–
<25	63	–	90.5% (95% CI, 80.4–96.4%)	–	–	
<20	36	–	100% (95% CI, 90.3–100%)	–	–	–

AUC, area under the curve; ACC, accuracy; SN, sensitivity; SP, specificity; NPV, negative predictive value; PPV, positive predictive value.

**Figure 1:**
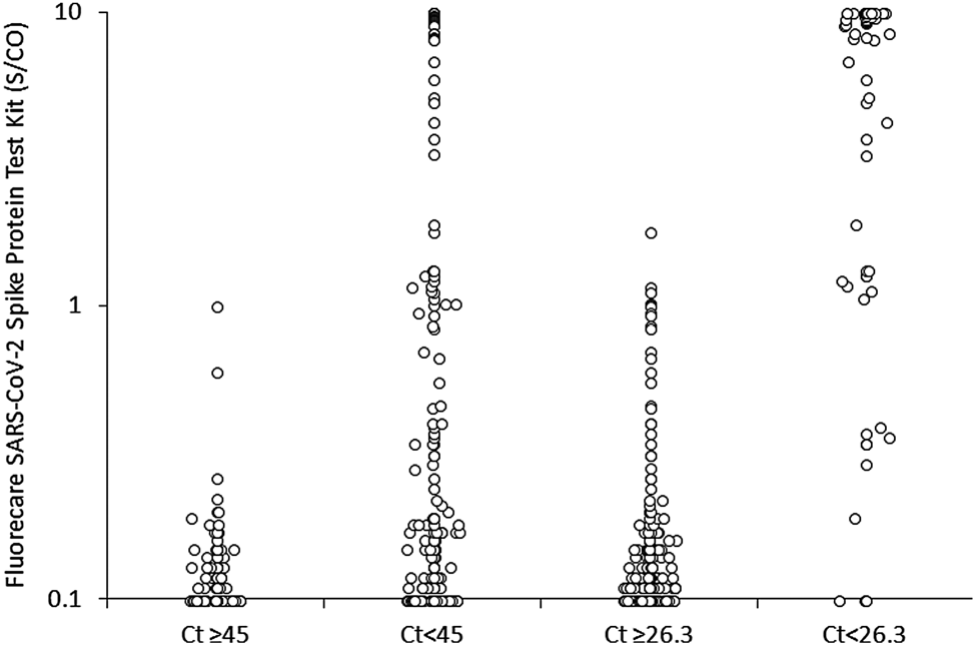
Distribution of Fluorecare SARS-CoV-2 Spike Protein Test Kit values according to the Altona thresholds for sample positivity (i.e., Ct values of both SARS-COV-2 *S* and *E* genes <45) and higher risk of infectivity (i.e., Ct values of both SARS-COV-2 *S* and *E* genes <26.3). Ct, cycle threshold; SARS-CoV-2, severe acute respiratory syndrome coronavirus 2; S/CO, signal to cut-off.

The distribution of Fluorecare SARS-CoV-2 Spike Protein Test Kit values according to the Altona thresholds for sample positivity (i.e., Ct values of both SARS-COV-2 *S* and *E* genes <45) or higher risk of infectivity (i.e., Ct values of both SARS-COV-2 *S* and *E* genes <26.3) is shown in Figure 1. The mean Fluorecare SARS-CoV-2 Spike Protein Test Kit values were found to be significantly higher in samples with Ct values <45 (n=233; 2.1 ± 3.6 S/CO) than in those with higher Ct values (n=121; 0.1 ± 0.1 S/CO; p<0.001), as well as in those with Ct values <26.3 (n=68; 6.7 ± 4.0 S/CO), than in those Ct values over this threshold (n=286; 0.2 ± 0.2 S/CO; p<0.001).

## Discussion

SARS-CoV-2 antigen testing, now mostly represented by Ag-RDT, is emerging as a possible alternative to overcome the dramatic burden posed on clinical laboratories by COVID-19 diagnostics [1, 4, 5]. A recent analysis of the Cochrane COVID-19 Diagnostic Test Accuracy Group has highlighted the theoretical advantages and possible drawbacks of these techniques [6]. As concerns the benefits, these tests are indeed simple, can be used easily by quickly trained staff or even by the patients themselves, do not necessitate centralized laboratory instrumentation and provide manageable results in a very short time. On the other hand, the clinical performance of Ag-RDTs is still considerably lower than that of molecular assay, and their cumulative diagnostic sensitivity remains limited at around 60–75% [6]. Especially modest performance has been reported in samples with low viral load (i.e., Ct values ≥25), with diagnostic sensitivity comprised between 30 and 50%, but increasing to 91–97% in samples with higher viral load (i.e., Ct values <25). Nevertheless, a rather broad heterogeneity of diagnostic performance of the different kits has been noted by the Cochrane COVID-19 Diagnostic Test Accuracy Group, with sensitivities ranging between dramatically low estimates (i.e., 12%), up to exceptionally high values (i.e., 90%, or even higher). This uncertain scenario has persuaded both the WHO and the IFCC Task Force on COVID-19 to recommend that each Ag-RDT should undergo a local validation process before being introduced into clinical practice [2], [3].

The results of our work have two major clinical implications. First, the relatively modest overall sensitivity and NPV of Fluorecare SARS-CoV-2 Spike Protein Test Kit would make it unadvisable to use this assay as alternative or even surrogate of conventional NAAT for precisely diagnosing SARS-CoV-2 infections. Nonetheless, its satisfactory sensitivity at higher nasopharyngeal viral load could make it a suitable test for screening patients with higher infective potential. A recent study, published by Gniazdowski et al. [7], found that the likelihood of obtaining a positive SARS-CoV-2 viral culture was <3% in nasopharyngeal samples with Altona Ct values <26.2. At a similar Ct threshold, Fluorecare SARS-CoV-2 Spike Protein Test Kit displayed a diagnostic sensitivity of around 90% in our cohort of unselected patients undergoing routine COVID-19 diagnostics, which would have permitted to identify the vast majority of individuals with high viral in the upper respiratory tract, who are those at the largest risk of spreading the virus in the community (i.e., the so-called super-spreaders), especially during mass gathering events [8].

Another important aspect to be considered, is that the nasopharyngeal viral load not only influence the likelihood of inter-individual SARS-CoV-2 transmission, but is also positively associated with the risk of progression towards severe/critical COVID-19 illness and/or death, as already demonstrated by several previous studies [9], [10], [11]. Therefore, rapid and efficient identification and/or monitoring of COVID-19 patients bearing high nasopharyngeal SARS-CoV-2 load during triage (even outside healthcare facilities), as well as during hospital stay or intensive care unit admission, would enable to establish a more timely and aggressive treatment, thus enhancing the likelihood of a positive outcome of this life-threatening disease.

In conclusion, our findings would hence suggest reserving the use of this Ag-RDT for identifying patients with higher viral load.
